# Increased Within-Network Functional Connectivity May Predict NEDA Status in Fingolimod-Treated MS Patients

**DOI:** 10.3389/fneur.2021.632917

**Published:** 2021-03-05

**Authors:** Claudia Piervincenzi, Nikolaos Petsas, Laura De Giglio, Maurizio Carmellini, Costanza Giannì, Silvia Tommasin, Carlo Pozzilli, Patrizia Pantano

**Affiliations:** ^1^Department of Human Neurosciences, Sapienza University of Rome, Rome, Italy; ^2^Department of Radiology, IRCCS NEUROMED, Pozzilli, Italy; ^3^Neurology Unit, San Filippo Neri Hospital, Rome, Italy; ^4^Multiple Sclerosis Center, S. Andrea Hospital, Department of Human Neurosciences, Sapienza University of Rome, Rome, Italy

**Keywords:** multiple sclerosis, resting-state functional MRI, functional connectivity, fingolimod, no evidence of disease activity

## Abstract

Only a few studies have evaluated the brain functional changes associated with disease-modifying therapies (DMTs) in multiple sclerosis (MS), though none used a composite measure of clinical and MRI outcomes to evaluate DMT-related brain functional connectivity (FC) measures predictive of short-term outcome. Therefore, we investigated the following: (1) baseline FC differences between patients who showed evidence of disease activity after a specific DMT and those who did not; (2) DMT-related effects on FC, and; (3) possible relationships between DMT-related FC changes and changes in performance. We used a previously analyzed dataset of 30 relapsing MS patients who underwent fingolimod treatment for 6 months and applied the “no evidence of disease activity” (NEDA-3) status as a clinical response indicator of treatment efficacy. Resting-state fMRI data were analyzed to obtain within- and between-network FC measures. After therapy, 14 patients achieved NEDA-3 status (hereinafter NEDA), while 16 did not (EDA). The two groups significantly differed at baseline, with the NEDA group having higher within-network FC in the anterior and posterior default mode, auditory, orbitofrontal, and right frontoparietal networks than the EDA. After therapy, NEDA showed significantly reduced within-network FC in the posterior default mode and left frontoparietal networks and increased between-network FC in the posterior default mode/orbitofrontal networks; they also showed PASAT improvement, which was correlated with greater within-network FC decrease in the posterior default mode network and with greater between-network FC increase. No significant longitudinal FC changes were found in the EDA. Taken together, these findings suggest that NEDA status after fingolimod is related to higher within-network FC at baseline and to a consistent functional reorganization after therapy.

## Introduction

Multiple sclerosis (MS) is an immune-system-mediated disease characterized by inflammation, demyelination, and degeneration of the central nervous system (CNS), leading to a deterioration in life quality and expectancy (1). Disease-modifying therapies (DMTs) have multiplied in the last 2 decades, with new categories of more targeted and effective drugs (2). Clinical trials have proven the efficacy of these treatments for relapsing MS in terms of clinical relapse rate, disability progression, and MRI-assessed lesion accumulation (3).

Multiple advanced MRI techniques have been used to investigate the mechanisms of action and the efficacy of DMTs in MS. Progress has also been made in the investigation of DMT effects on the brain in terms of tissue loss, microstructural abnormalities, metabolic changes, and functional plasticity [for a review, see (4)]. Over the years, functional MRI (fMRI) has provided important insights into MS-damage-related plasticity and functional reorganization, although only a few studies have evaluated brain functional changes associated with DMTs in MS. Of these, 2 studies used motor-task-evoked fMRI to investigate therapy-related (IFN-β 1a) functional activity changes in the sensorimotor network (5, 6). Even less is known about resting-state functional connectivity (FC) changes after DMT. With respect to task-based fMRI, resting-state fMRI is more advantageous in clinical studies because it is easily applicable, allows the simultaneous investigation of different brain networks, and avoids the confounding effects of disability in performing a given behavioral task (7, 8).

In our recent study, we investigated FC changes of the primary motor cortex in relapsing MS after 6 months of fingolimod treatment (Gilenya®; Novartis) (9). Using a seed-based approach, we found a significant decrease in FC between this region and posterior cortical areas, which correlated with a significant cognitive improvement as measured by the Paced Auditory Serial Addition Task (PASAT). However, in that study, we directly explored changes only in the sensorimotor network through a seed-based analysis of the primary motor cortex. The other resting-state networks (RSNs), including those directly cognition related, were not investigated as that would have been beyond the scope of the study. Moreover, treatment efficacy was measured in terms of conventional MRI parameters (gadolinium-positive lesions and new T2 lesions), although the attempt to use them as grouping criterion did not lead to significant results. Thus, our previous results have suggested the need to investigate large-scale network connectivity to better understand DMT-related brain functional rearrangement and FC measures that predict treatment outcome. Therefore, the present study aims to (1) identify baseline FC differences between patients who showed evidence of disease activity after a specific DMT and those who did not; (2) investigate the longitudinal effect of DMT on FC among responders and non-responders, and (3) identify possible relationships between FC changes and DMT-related changes in performance.

For this purpose, we used the dataset from our previous study (9) on relapsing MS patients who underwent fingolimod treatment for 6 months. After that period of time, we used the “no evidence of disease activity” (NEDA-3) status characterization as a clinical response indicator of treatment efficacy (2, 10). We hypothesized that FC before and during treatment reflects a brain status that would determine susceptibility to DMT action on the basis of neuroinflammatory and/or neuroplasticity mechanisms. In this framework, NEDA status achievement would be reflected in DMT-induced functional reorganization at the level of large-scale brain networks joined to the anti-inflammatory effect of DMT. Furthermore, we also hypothesized that the clinical relevance of FC changes would be reflected in their correlation with concomitant changes in behavioral measures. To confirm this hypothesis, we expected the following outcomes: (1) prediction of NEDA status based on baseline FC and (2) a correlation between FC longitudinal changes after 6 months of DMT and significantly changed behavioral measures.

## Materials and Methods

### Participants

All patients belonged to a previously published prospective, post-marketing, interventional study [for details, see (9)], in which a consecutive series of 36 patients with a diagnosis of relapsing-remitting MS (11) were recruited at Sant'Andrea Hospital, Sapienza University of Rome, Italy and underwent an MRI scan and clinical testing at Policlinico Umberto I, Sapienza University of Rome, Italy. Thirty-two patients completed the protocol and were included in the previous analyses; this cohort constituted our initial dataset. We analyzed MRI images acquired 1–2 days prior to starting fingolimod [therapy start (Tst)] and after 6 months of fingolimod treatment (T6m). Two patients were excluded from the present study because of a missing postgadolinium T1 sequence. Of the remaining 30 patients, 4 were treatment naïve, 20 switched from a previous first-line, and 6 switched from a second-line DMT. The switch from first-line DMT was due to inefficacy while that from the second line (Natalizumab) was due to Progressive Multifocal Leukoencephalopathy risk. Patients were then divided into groups according to the NEDA-3 composite outcome measure (10). Patients who achieved NEDA-3 status (hereinafter referred to as NEDA) were defined as those who had: (1) no relapses, (2) no confirmed disability progression at 6 months, and (3) no new/enlarged T2 or gadolinium positive lesions. Patients who did not achieve NEDA were defined as EDA.

At each time point and before being scanned, participants were assessed by a trained physician and the following clinical outcomes were collected: Expanded Disability Status Scale (EDSS) score (12), MS Functional Composite (MSFC) (13) score with its subscores [9-Hole Peg Test (9-HPT); 25-Feet Walk Test (25-FWT); and the Paced Auditory Serial Addition Test (PASAT) 3 and 2 s].

### Ethics Statement

This study was performed in accordance with the ethical code of the ethics committee of Azienda Policlinico Umberto I, Sapienza University of Rome, and the Declaration of Helsinki. After approval from the ethics committee, written informed consent was obtained from all subjects.

### MRI Acquisition

Images were acquired with a 3-Tesla (3T) scanner (Siemens Magnetom Verio) and a 12-channel head coil designed for parallel imaging (GRAPPA). Participants were advised to avoid consuming psychoactive substances, such as tea or coffee, within 2 h prior to MRI scans.

The following sequences were acquired:

- Blood oxygen level-dependent (BOLD) single-shot echo-planar imaging (TR = 3,000 ms, TE = 30 ms, flip angle = 89°, FOV = 192 mm, 64 × 64 matrix, 50 contiguous axial slices 3-mm thick, 140 volumes, voxel size = 3 mm^3^, acquisition time = 7 min 11 s), with all patients instructed to close their eyes and stay awake during the resting-state fMRI acquisitions;
- High-resolution 3D, T1-weighted (T1-3D) MPRAGE sequence (TR = 1,900 ms, TE = 2.93 ms, TI = 900 ms, flip angle = 9°, FOV = 260 mm, matrix = 256 × 256, 176 sagittal slices 1 mm thick, no gap);
- Dual turbo spin-echo, proton density (PD) and T2-weighted images (TR = 3,320 ms, TE1 = 10 ms, TE2 = 103 ms, FOV = 220 mm, matrix = 384 × 384, 25 axial slices 4 mm thick, 30% gap);
- T1-weighted spin-echo sequence acquisition after administration of a gadolinium-based contrast agent (TR = 550 ms, TE = 9.8 ms, FOV = 240 mm, matrix = 320 × 320, 25 axial slices 4 mm thick, 30% gap).

### MRI Analysis

#### Data Quality

Image quality metrics were extracted from functional and structural MRI data using the default pipeline of MRIQC (14), an open-source analysis program that estimates image quality metrics (IQMs) to provide interoperability, uniform standards, and to assess reliability of a dataset. Supplementary Tables 1, 2 provide lists of all IQMs obtained with MRIQC, showing that our measures are in line with those of other studies that have provided IQMs (e.g., see http://mriqc.org).

#### Lesion Load and Brain Volume

Lesion volume was calculated on the PD images using a semi-automated technique with Jim 5.0 software (Xinapse System, Leicester, UK; http://www.xinapse.com). Measures of global brain volume and gray matter (GM) volume at baseline (i.e., at Tst) were also obtained from lesion-filled T1-3D brain images *via* SIENAX software, a fully automated method for measuring cross-sectional brain volume that is freely available as part of FMRIB's Software Library (FSL) (https://fsl.fmrib.ox.ac.uk/fsl/fslwiki).

### Functional Connectivity

#### Data Pre-processing

Functional MRI data were preprocessed using FSL, version 5.0.9. Single-subject preprocessing was performed using FMRI Expert Analysis Tool (FEAT). Pre-statistical processing consisted of motion correction using MCFLIRT (15), brain extraction using BET (16), slice-timing correction, spatial smoothing using a Gaussian kernel of full-width at half-maximum of 5 mm, and high-pass temporal filtering with a cut-off of 100 s. Registration to high-resolution structural and/or standard space images was performed using FLIRT (15, 17). EPI volumes were registered to the individual structural scan using the FLIRT_Boundary-Based Registration (BBR) tool (18). Registration from high-resolution structural to standard space was then further refined using FNIRT non-linear registration (19). 4D GM maps were obtained using the *feat_gm_prepare* tool and were used as voxel-wise nuisance variables in subsequent statistical analyses.

Independent component analysis (ICA) of preprocessed functional data was performed using Multivariate Exploratory Linear Optimized Decomposition into Independent Components (MELODIC) tool (20). For group-wise ICA, a single 4D dataset was created by temporally concatenating preprocessed functional data containing 140 time points for each subject. The dimensionality of group-ICA was performed using different numbers of components (i.e., 20, 25, 30, 35, 40) (20–22). Finally, a dimensionality of 25 was chosen, as the explained data variance (45.95%) was sufficient to obtain good estimates of the signals, and well-known RSNs were identified (21). RSNs of interest were identified via spatial correlation coefficients (*fslcc* tool) using RSNs generated by Smith et al. (21) and Yeo et al. (23) as templates, and then verified by expert visual inspection (CPi, NP, PP).

The set of spatial maps from the group-average analysis was used to generate subject-specific versions of the spatial maps and associated time series using a dual regression technique (24, 25). For each subject, the group-average set of spatial maps was first regressed (as spatial regressors in a multiple regression) into the subject's 4D space-time dataset, resulting in a set of subject-specific time series, one per group-level spatial map. These time series were then regressed (as temporal regressors in a multiple regression) into the same 4D dataset, resulting in a set of subject-specific spatial maps, one per group-level spatial map. Individual difference maps between T6m and Tst (ΔFC maps) were also obtained for each RSN.

### Statistical Analyses

Statistical analyses of demographic, clinical, radiological, and neuropsychological parameters were performed using SPSS statistics software (version 22.0). To investigate the possible confounding role of the above parameters in the prediction of NEDA status, between-group differences at Tst were tested using Mann–Whitney *U*-test and Chi-square test for continuous and dichotomous variables, respectively (*p* < 0.05 for null hypothesis rejection). In the subgroup of interest (NEDA), longitudinal changes in the neuropsychological scores were estimated using Wilcoxon signed-rank test (*p* < 0.05 for null hypothesis rejection); for significant changes, individual differences between T6m and Tst (Δ) were obtained and used for voxel-wise correlation with ΔFC maps.

#### Head Motion Analysis

Head motion (even if small) can bias derived results in groups where there are motion differences (26–28). To examine if there are any differences in head motion among NEDA and EDA patients at baseline, or between sessions for each subgroup, absolute and relative displacement values were obtained by using the McFLIRT tool in FSL (15) and compared using a two-sample unpaired *t*-test and paired *t*-test, respectively, with *p* < 0.05 for null hypothesis rejection.

#### Within-Network Functional Connectivity

Subject-specific spatial maps obtained from dual regression analysis were entered into group-level voxel-wise analyses. To investigate within-network FC differences at Tst, we compared the 2 patient subgroups using a two-sample unpaired *t* test.

To investigate within-network FC changes after 6 months of treatment and compare longitudinal changes between the 2 patient subgroups, a two-sample unpaired *t*-test was performed on ΔFC maps of NEDA and EDA patients. Age, sex, and GM maps were entered as nuisance variables in all analyses.

Voxel-wise statistical analyses were performed with permutation-based non-parametric statistics using FSL Randomize permutation-based program with 5,000 permutations (29). Clusters were determined by using threshold-free cluster enhancement (TFCE) (30) and a family-wise error (FWE)-corrected cluster significance threshold of *p* < 0.05. The Randomize tool (5,000 permutations) was also used to examine the statistical correlation between significant within-network FC longitudinal changes and behavioral parameters that changed over time. Correlation analyses were performed inside the mask of significant longitudinal FC changes. Resulting statistical maps were thresholded at *p* < 0.05, FWE corrected. Anatomical localization of significant clusters was established according to the Harvard-Oxford Cortical Structural Atlas included in FSL (http://www.fmrib.ox.ac.uk/fsl/data/atlas~descriptions.html).

#### Between-Network Functional Connectivity

Between-network functional connectivity differences were investigated using FSL Nets toolbox (http://fsl.fmrib.ox.ac.uk/fsl/fslwiki/FSLNets). After normalization of the extracted time courses (output of the first stage of dual regression) of all RSNs identified in each subject, time courses of artifactual components and components of no interest were regressed out of the individual data. Subject-wise correlation matrices of both full and partial correlation of all remaining RSN time courses were then created. Resulting correlation coefficients (connection or edge strengths) were then Fisher z-transformed and corrected for temporal autocorrelation. Between-subject testing was then conducted across correlation values acquired for pairs of independent components.

Between-network connectivity differences at Tst between the 2 patient subgroups were investigated using non-parametric unpaired testing (FSL Randomize tool, 5,000 permutations; age, sex, and GM as nuisance variables). Between-network connectivity differences between Tst and T6m were investigated using non-parametric paired testing. The statistical significance threshold was set at *p* < 0.05, FWE corrected. Finally, the relationship between behavioral changes and between-network FC changes (ΔFC, expressed as the difference between T6m and Tst of the z-transformed correlation coefficients) was assessed using Spearman's rank correlation in SPSS, with *p* < 0.05 for null hypothesis rejection, corrected for multiple comparisons.

## Results

### Clinical and Conventional MRI Findings

Descriptive statistics for demographic, clinical, radiological, and neuropsychological parameters at Tst in the 2 patient subgroups are reported in Table 1.

**Table 1:** Demographic and clinical characteristics, radiological features, and scores obtained in the clinical/neuropsychological assessment at fingolimod therapy start (Tst) in the 2 patient subgroups.

	**NEDA (*N* = 14)**	**EDA (*N* = 16)**	p^*^
**Demographic/clinical features**			
Age	37.4 ± 6.5	36.1 ± 8.4	ns
Female/male (*n*)	8/6	15/1	0.018
Disease duration (year)	10.4 ± 5.6	10.0 ± 8.3	ns
EDSS score [median (range)]	1.75 [1.5–5.0]	2.0 [1.0–6.0]	ns
Relapses in previous year [*n* (%)]	4 (29)	9 (56)	ns
Treatment naïve [*n* (%)]	3 (21)	1 (6)	ns
First-line/second-line therapy (*n*)	8/3	12/3	ns
**Radiological features**			
Brain volume (cm^3^)	1,464 ± 68	1,495 ± 66	ns
Gray matter volume (cm^3^)	735 ± 48	765 ± 39	ns
T2-lesion volume (cm^3^)	14.239 ± 12.143	6.653 ± 4.979	ns
Gadolinium-positive lesion [*n* (%)]	5/14 (36)	10/16 (62)	ns
**Clinical/Neuropsychological scores**			
9-HPT dominant hand (s)	20.4 ± 4.9	19.1 ± 3.0	ns
9-HPT non-dominant hand (s)	22.3 ± 7.4	20.8 ± 4.0	ns
PASAT 3 (s)	42.5 ± 13.7	45.4 ± 12.8	ns
PASAT 2 (s)	35.1 ± 15	35.7 ± 12.9	ns
25-FWT (s)	5.5 ± 1.2	6.5 ± 2.1	ns

*Values are reported as the mean ± standard deviation or median [min-max]*.

*n, number; s, seconds; ns, not statistically significant*.

* *Between-group differences at Tst were tested using Chi-square test (sex, treatment-naïve dichotomous variables) and Mann–Whitney U-test (all other continuous variables) (p < 0.05 for null hypothesis rejection)*.

After 6 months of therapy, 14 patients achieved NEDA (46.65%), while 16 did not (53.35%). In the EDA subgroup, 9 patients had MRI activity, 4 had both clinical and MRI activity, and 3 had disability worsening.

There were no significant differences at Tst (Table 1) between NEDA and EDA in terms of age, disease duration, EDSS score, number of relapses in the previous year, number of treatment-naïve patients, and number of patients switching from first- or second-line DMTs. Regarding radiological characteristics, no significant differences at Tst were found between the 2 subgroups in terms of brain volume, gray matter volume, lesion volume, and the number of gadolinium-enhancing lesions. No significant differences were found between NEDA and EDA patients in terms of neuropsychological scores. The only significant difference between subgroups was gender distribution, with 6 males in the NEDA subgroup vs. one in the EDA subgroup.

In NEDA patients, PASAT scores showed a significant longitudinal improvement after 6 months of therapy (PASAT3: *p* = 0.033; PASAT2: *p* = 0.046), while 9-HPT and 25-FWT scores did not show any significant longitudinal change.

### Resting-State Functional Connectivity

#### Head Motion

At Tst, maximum absolute head motion was 0.53 mm in the NEDA group and 0.87 mm in the EDA group. Mean absolute and relative displacement values were 0.24 ± 0.11 and 0.05 ± 0.02 mm in NEDA, 0.25 ± 0.19 and 0.04 ± 0.01 mm in EDA. At T6m, maximum absolute head motion was 0.55 mm in the NEDA group and 0.40 mm in the EDA group. Mean absolute and relative displacement values were 0.29 ± 0.16 and 0.05 ± 0.02 mm in NEDA and 0.22 ± 0.08 and 0.03 ± 0.01 mm in EDA. No subject was identified as a motion outlier. There were no significant differences in motion parameters between NEDA and EDA at baseline, nor between sessions in each subgroup.

#### Within-Network Connectivity

**Figure 1:**
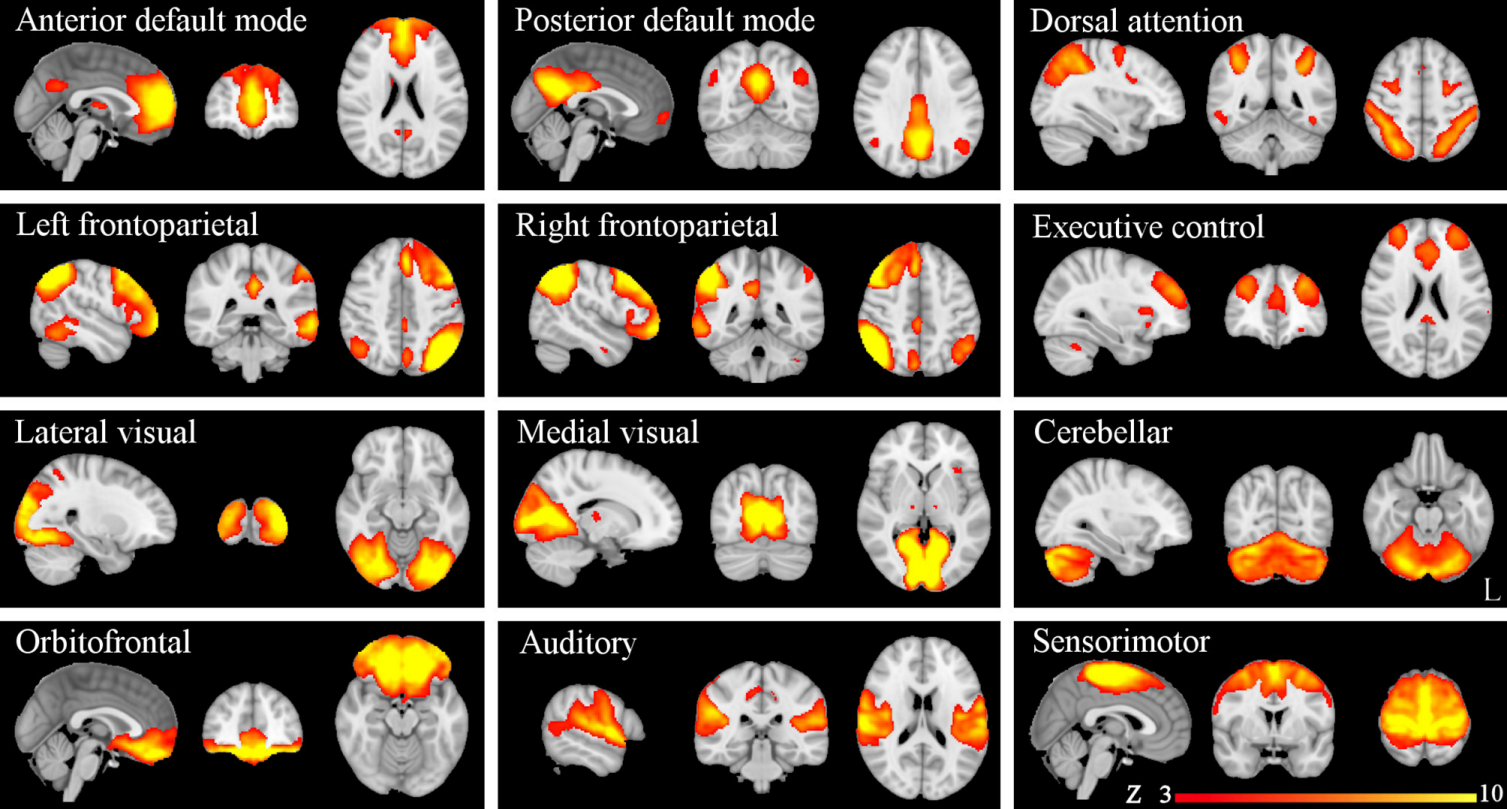
Resting-state networks (RSNs) identified and used for dual regression analysis. This figure shows sagittal, coronal, and axial slices for the RSNs detected, overlaid onto the MNI152 standard brain. RSNs are shown in FSL red-yellow color encoding using a 3 < z-score < 10 threshold window.

ICA yielded 25 independent components representing group-averaged networks of brain regions with BOLD fMRI signals that were temporally correlated. Of these, we identified 12 components that showed the highest spatial correlation coefficients with RSN templates, accounting for 49.9% of the explained variance and for 22.92% of the total variance: the anterior and posterior default mode (*r* = 0.41 and *r* = 0.67, respectively), dorsal attention (*r* = 0.51), left and right frontoparietal (*r* = 0.68 and *r* = 0.67, respectively), executive control (*r* = 0.52), lateral visual (*r* = 0.65), medial visual (*r* = 0.76), cerebellar (*r* = 0.30), orbitofrontal (*r* = 0.40), auditory (*r* = 0.69), and sensorimotor (*r* = 0.58) networks (Figure 1).

**Figure 2:**
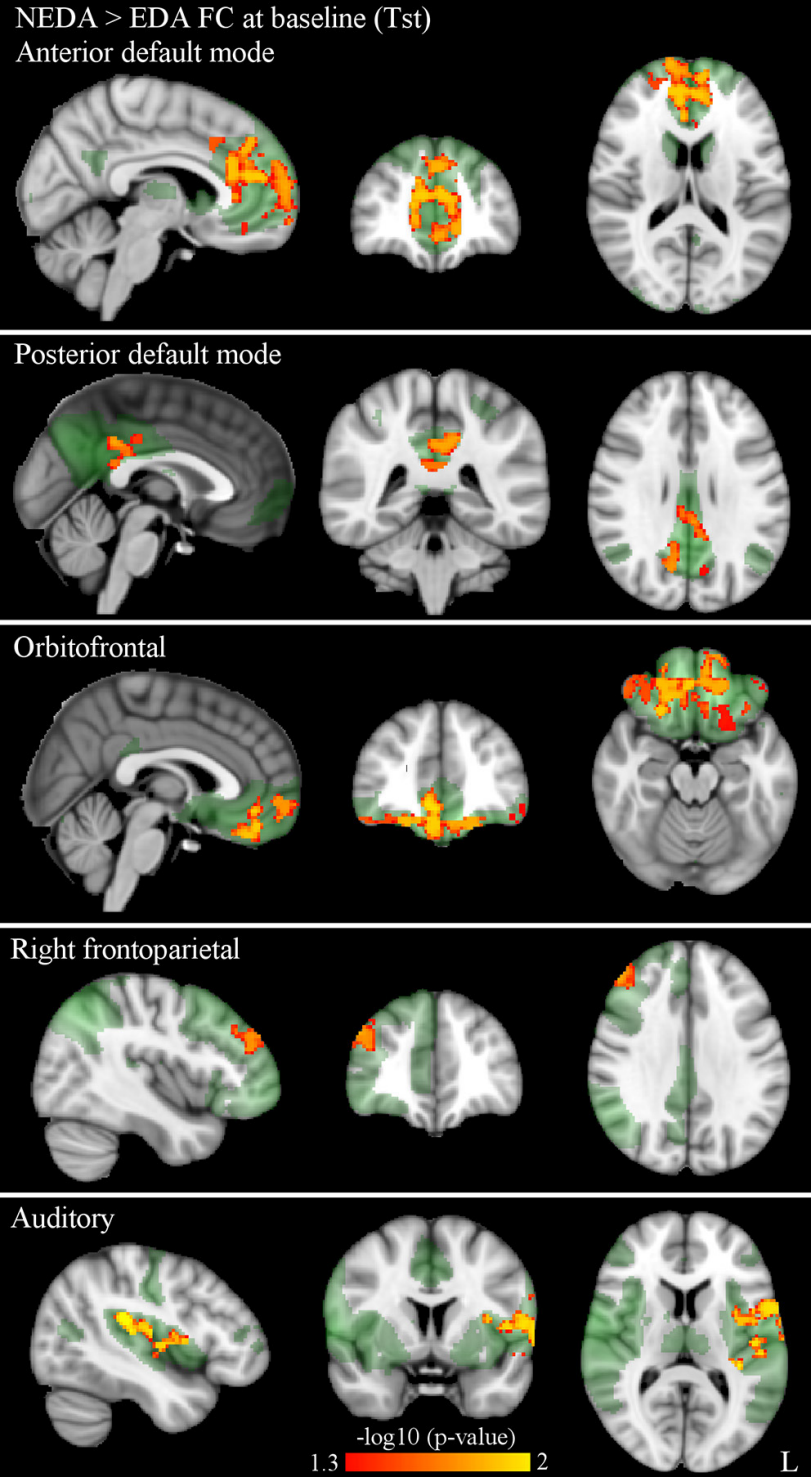
Significantly higher within-network FC in NEDA as compared with EDA patients at baseline in the anterior default, posterior default, orbitofrontal, right frontoparietal, and auditory networks (*p* < 0.05, FWE corrected). Results for each RSN are overlaid onto the corresponding network (green) in the MNI152 standard brain. The red-yellow color bar represents level of significance. The –log10(*p*)-value represents the negative base-10 logarithm of the *p* value. A larger value of –log10(*p*) corresponds to a smaller *p*-value.

At Tst, significant differences in within-network FC were found between patient subgroups. More specifically, NEDA patients showed significantly higher FC with respect to EDA patients in five RSNs, namely the anterior and posterior default mode, orbitofrontal, right frontoparietal, and auditory networks (*p* < 0.05, FWE corrected) (Figure 2; Table 2).

**Table 2:** RSNs showing significantly higher within-network FC in NEDA as compared with EDA patients at baseline (*p* < 0.05, FWE corrected).

		**MNI coordinates**			
**Cluster size**	***p***	***x***	***y***	***z***	**Cluster location (local maxima)**
**(voxels)**					
**Anterior default mode network**					
2,915	0.014	8	46	16	Paracingulate gyrus
	0.015	12	66	12	Right frontal pole
	0.015	−10	56	10	Left frontal pole
	0.018	−2	44	−8	Paracingulate gyrus
	0.024	−10	42	34	Left superior frontal gyrus
**Posterior default mode network**					
871	0.018	−4	−42	32	Cingulate gyrus, posterior division
	0.019	12	−66	26	Precuneous cortex
	0.035	14	−34	38	Cingulate gyrus, posterior division
42	0.047	−14	−60	18	Precuneous cortex
39	0.047	−8	−68	24	Precuneous cortex
**Auditory network**					
1,465	0.01	−44	−30	18	Left parietal operculum cortex
	0.012	−56	6	6	Left precentral gyrus
	0.013	−42	−10	2	Left insular cortex
**Orbitofrontal network**					
1,868	0.011	6	36	−10	Paracingulate gyrus
	0.014	20	22	−24	Right frontal orbital cortex
	0.014	24	52	−12	Right frontal pole
	0.016	−18	42	−22	Left frontal pole
	0.02	4	56	−14	Frontal pole/frontal medial cortex
62	0.046	−48	44	−12	Left frontal pole
**Right frontoparietal network**					
263	0.017	48	38	32	Right frontal pole/middle frontal gyrus

*Peak MNI coordinates (mm) within clusters were identified using the minimum peak distance between the local maxima of 20 mm. Anatomical localizations of peak MNI coordinates were established according to the Harvard-Oxford Cortical Structural Atlas*.

**Figure 3:**
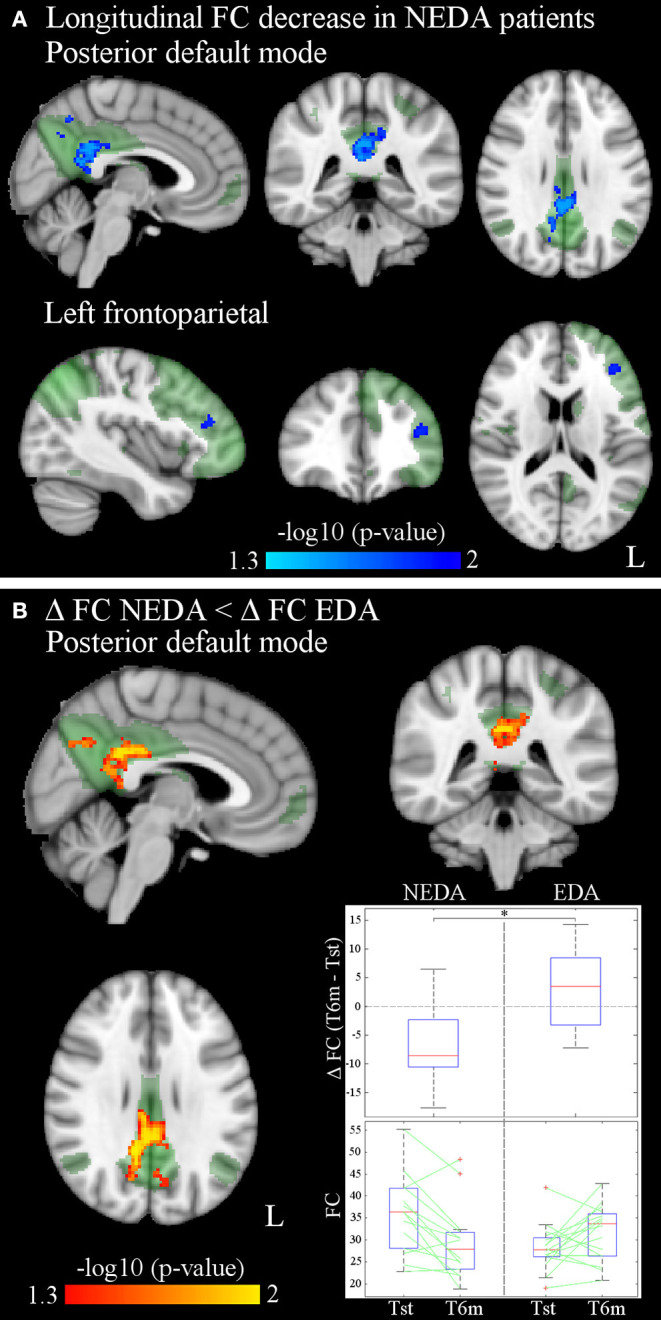
**(A)** Significantly decreased within-network FC of the posterior default mode and left frontoparietal networks after 6 months of fingolimod in NEDA patients (*p* < 0.05, FWE corrected). Results for each RSN are overlaid onto the corresponding network (green) in the MNI152 standard brain. The blue/light-blue color bar represents level of significance. The –log10(*p*)-value represents the negative base-10 logarithm of the *p*-value. A larger value of –log10(*p*) corresponds to a smaller *p*-value. **(B)** Significant differences between ΔFC in NEDA and EDA patients (*p* < 0.05, FWE corrected): ΔFC in NEDA is significantly lower than ΔFC in EDA patients. The red-yellow color bar represents level of significance. The box plots show the distribution of ΔFC (T6m-Tst) values of NEDA and EDA patients, and the individual values at Tst and T6m for each group inside the mask of between-group differences. On each box, the central mark indicates the median, and the bottom and top edges of the box indicate the 25th and 75th percentiles, respectively. The whiskers extend to the most extreme data points not considered outliers, and the outliers are plotted individually using the “+” symbol.

After 6 months of therapy, decreased within-network FC was found in NEDA patients in regions of the posterior default mode and left frontoparietal networks (*p* < 0.05, FWE corrected) (Figure 3A; Table 3). No significant FC changes were found in EDA patients after therapy. The comparison between ΔFC maps of NEDA and EDA patients showed a significant difference in the posterior default mode network (*p* < 0.05, FWE corrected), with NEDA patients showing a significant reduction in FC over time as compared with the EDA group which showed no significant change (Figure 3B; Table 4).

**Table 3:** RSNs showing significant within-network FC reduction after 6 months of fingolimod therapy in NEDA patients (*p* < 0.05, FWE corrected).

		**MNI coordinates**			
**Cluster size (voxels)**	***p***	***x***	***y***	***z***	**Cluster location (local maxima)**
**Posterior default mode network**					
656	0.018	2	−42	28	Cingulate gyrus, posterior division
	0.028	10	−60	34	Precuneous cortex
	0.042	−12	−28	36	Cingulate gyrus, posterior division
**Left frontoparietal network**					
52	0.038	−40	38	18	Left frontal pole/middle frontal gyrus

*Refer to Table 2 for a detailed explanation of the table layout*.

**Table 4:** Significant within-network FC differences between ΔFC maps of NEDA and EDA patients in the posterior default mode network (*p* < 0.05, FWE corrected).

		**MNI coordinates**			
**Cluster size (voxels)**	***p***	***x***	***y***	***z***	**Cluster location (local maxima)**
**Posterior default mode network**					
987	0.011	10	−48	26	Cingulate gyrus, posterior division
	0.020	−10	−54	34	Precuneous cortex
	0.025	6	−72	32	Precuneous cortex

*ΔFC in NEDA patients was significantly lower than ΔFC in EDA patients. Refer to Table 2 for a detailed explanation of the table layout*.

**Figure 4:**
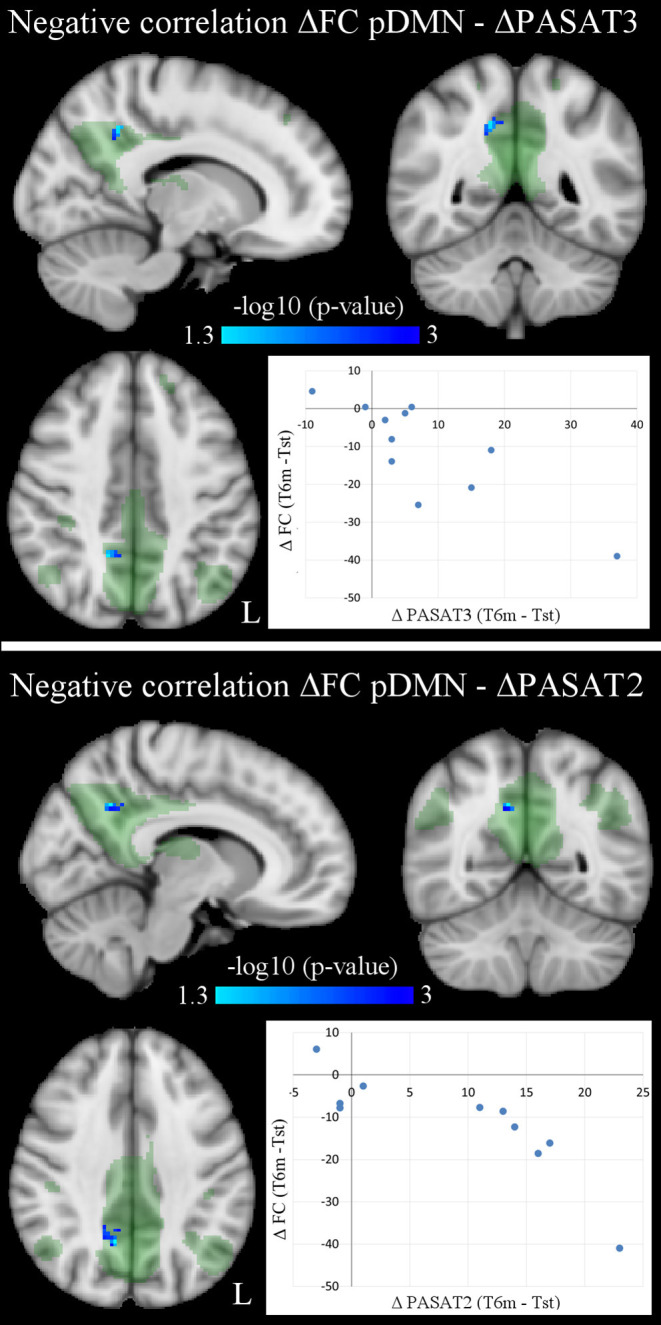
Voxel-wise negative correlations in NEDA patients between within-network FC changes in posterior default mode network (pDMN) and PASAT improvement (ΔPASAT3 and ΔPASAT2) after 6 months of fingolimod (*p* < 0.05, FDR corrected). As shown in the scatterplots, the greater the PASAT increase, the greater the FC decrease (ΔFC values obtained at cluster local maxima). Results are overlaid onto the posterior default mode network map (green) in the MNI152 standard brain. The blue/light-blue color bar represents level of significance. The –log10(*p*)-value represents the negative base-10 logarithm of the *p*-value. A larger value of –log10(*p*) corresponds to a smaller *p*-value.

Correlation analyses between significant longitudinal FC changes and behavioral changes in NEDA patients yielded no FWE-corrected results. However, since the number of NEDA patients was low and our previous study on the same cohort already reported a significant correlation between therapy-related FC changes and cognitive performance (9), statistical results using false-discovery rate (FDR) multiple-comparisons correction (*p* < 0.05) are also reported (Figure 4; Table 5). Voxel-wise negative correlations were found in NEDA patients between FC decrease in the posterior default mode network and increase in ΔPASAT3 and ΔPASAT2 after therapy. The greater the PASAT increase at 6 months, the greater the FC decrease in the posterior default mode network. No significant correlations were found with the left frontoparietal network.

**Table 5:** Significant negative correlations between longitudinal FC changes in the posterior default mode network (ΔpDMN) and PASAT improvements (ΔPASAT3 and ΔPASAT2) in NEDA patients (*p* < 0.05, FDR corrected).

		**MNI coordinates**			
**Cluster size (voxels)**	***p***	***x***	***y***	***z***	**Cluster location (local maxima)**
**ΔpDMN-ΔPASAT3 ↓**					
22	0.001	14	−50	42	Precuneous cortex
**ΔpDMN-ΔPASAT2 ↓**					
46	<0.001	10	−56	36	Precuneous cortex

*Refer to Table 2 for a detailed explanation of the table layout*.

To address the predictive value of baseline FC for NEDA status, *post-hoc* logistic regression analyses were performed using SPSS to ascertain the effect of FC on the likelihood of achieving NEDA. In the statistical model, within-network FC was represented by the mean value inside the mask of each RSN, showing a significant connectivity difference between NEDA and EDA patients (auditory, anterior default mode, posterior default mode, orbitofrontal, and right frontoparietal networks). We firstly investigated correlations between within-network FC values of the 5 RSNs and found them significantly correlated (*p* < 0.05). Therefore, to avoid multicollinearity issues, we run the logistic regression including each RSN one by one. The logistic regression models were statistically significant for each RSN investigated (*p* < 0.05), explaining a percentage of variance (Nagelkerke *R*^2^) in NEDA status ranging from 36 to 79%, and correctly classifying 80–93% of cases.

#### Between-Network Connectivity

**Figure 5:**
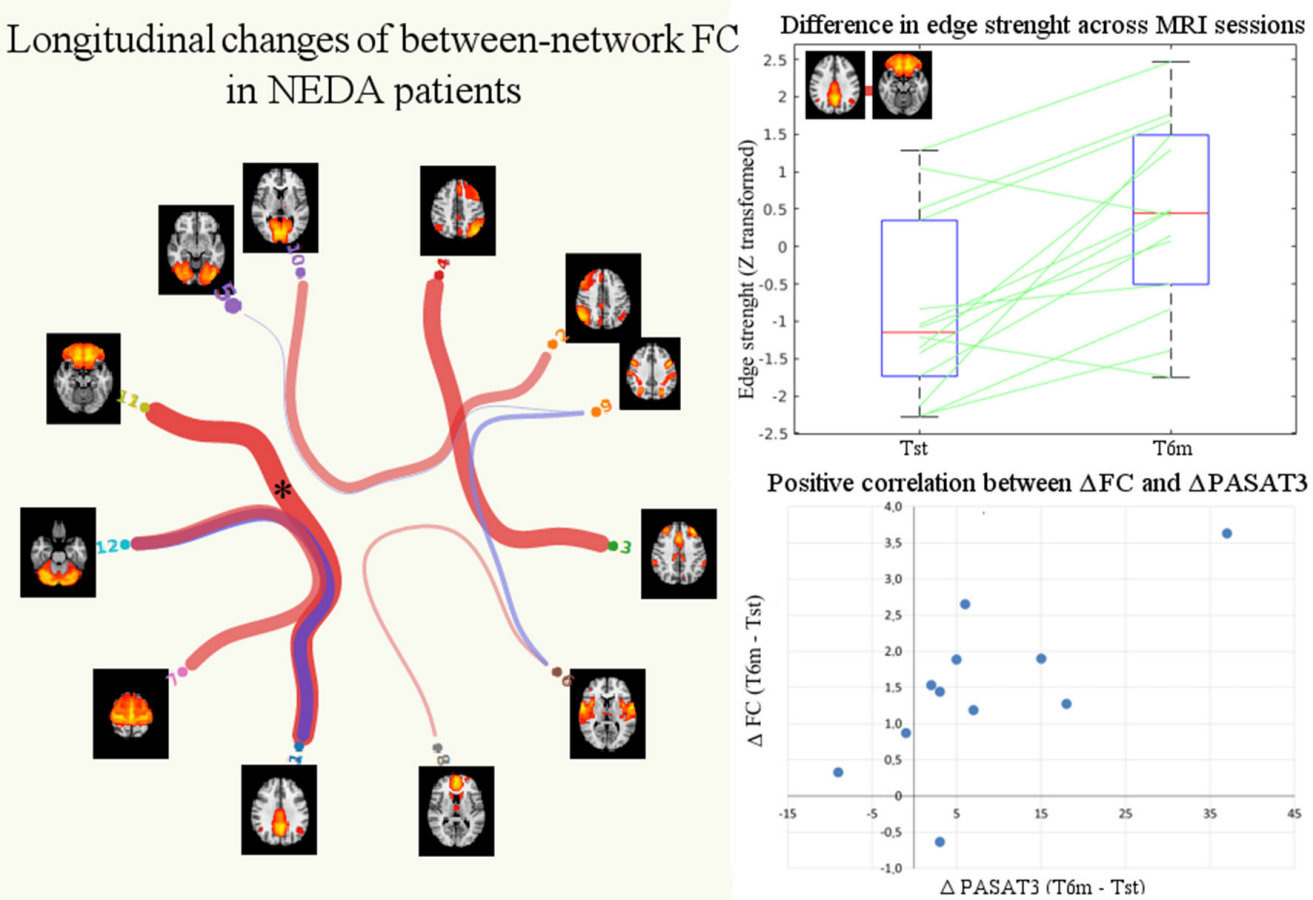
Significant changes in between-network FC in NEDA patients after 6 months of fingolimod. The NEDA subgroup showed a significantly higher correlation between the posterior default mode and orbitofrontal networks (*p* < 0.05 FWE corrected, indicated with asterisk). The box plot shows the distributions of the correlation values (edge strengths) at Tst and T6m in NEDA patients. On each box, the central mark indicates the median, and the bottom and top edges of the box indicate the 25th and 75th percentiles, respectively. The whiskers extend to the most extreme data points. The scatterplot shows the positive correlation between posterior default mode/orbitofrontal networks increased FC (ΔFC, expressed as the difference between T6m and Tst of the z-transformed correlation coefficients) and PASAT improvement (ΔPASAT3).

No significant differences in partial or full correlations between RSNs were found at Tst between NEDA and EDA patients. Instead, after therapy NEDA patients showed a significantly higher partial correlation between the posterior default mode and orbitofrontal networks (*p* < 0.05, FWE corrected) (Figure 5). Moreover, a positive correlation was found between ΔPASAT3 and FC changes in the posterior default mode network/orbitofrontal network correlation (Spearman rho = 0.61, *p* = 0.048); the greater the PASAT improvement, the greater the FC increase (Figure 5). No significant differences in partial or full correlations between RSNs were found in EDA patients after therapy.

## Discussion

In the present postmarketing study, we analyzed data from a previously published dataset on relapsing MS patients who underwent fingolimod treatment for 6 months (9), using the NEDA-3 status characterization as a clinical response indicator of treatment efficacy.

We found significant baseline differences in within-network FC between NEDA and EDA patients in several RSNs, with NEDA patients showing higher FC than EDA patients. Furthermore, only NEDA patients showed significant changes in within- and between-network FC after fingolimod. These longitudinal changes mainly regarded the posterior default mode network and correlated with a significant improvement in PASAT score.

### Epidemiological Coherence

In our study, almost half of patients achieved NEDA after 6 months of treatment (46.6%). This result is consistent with real-world studies on large populations of relapsing MS patients receiving fingolimod that have reported that 35–60% of patients achieve NEDA-3 status over 1–2 years of treatment (31, 32). We did not find any significant differences in radiological or neuropsychological variables between NEDA and EDA patients at baseline. However, we found a significantly higher number of males in the NEDA group. Regarding treatment-by-sex interaction, literature shows inconsistent findings [see (33) for details] and the small number of males in the present study did not allow further generalization.

### Predictive Value of Baseline Functional Connectivity for Treatment Response

The search for predictive biomarkers in MS treatment is ongoing. In fingolimod treatment, some recent studies have found different baseline variables associated with a higher likelihood of achieving NEDA. Giuliani and colleagues (34) reported that baseline clinical factors, such as a higher baseline EDSS score and more relapses in the year before the start of fingolimod were highly associated with the risk of *not* achieving NEDA. Other authors have reported that baseline parameters of higher disease activity (annualized relapse rate, gadolinium-enhancing lesions, and age at onset) were associated with an increased likelihood of failing to achieve NEDA criteria (32).

This is the first study that investigates and reports significant baseline FC differences between patients who achieved NEDA and those who did not. Of note, no other radiological parameter at baseline differed between NEDA and EDA patients. Accordingly, with reference to the possible effect of an established DMT therapy, we can hypothesize a specific predictive value of baseline within-network FC for NEDA after a time lapse of 6 months.

### Functional Reserve Engagement as a Putative Mechanism for NEDA Prediction

We hypothesize that functional reserve could play a role in baseline differences. According to Medaglia et al. (35), “reserve represents individual variability in the functional use or structural integrity of the nervous system that alters a person's cognitive and behavioral abilities following the onset of brain pathology. In essence, the concept of reserve suggests that some initial conditions of brain physiology and function—often measured using neuroimaging techniques—heavily constrain the observed clinical sequelae.” Consistent with an increasing number of studies suggesting that RSN organization is associated with reserve level and reflects the influence of reserve on brain network organization (36), in our study, we found that a higher connectivity at baseline could characterize MS patients with a higher functional reserve. Indeed, previous studies have linked altered FC with reduced or preserved functional reserve in MS (37–39).

### Fingolimod Treatment Reduces Baseline High Functional Connectivity

At the longitudinal level, we found significant FC changes in NEDA patients, suggesting that pharmacological modulation promotes a more favorable reorganization only in those with a more efficient functional reserve. However, it is not fully understood how immunomodulation modifies recovery and connectivity. The anti-inflammatory and immunomodulatory effects of fingolimod may result in the restoration of synaptic plasticity and, in turn, the facilitation of recovery (40, 41).

Longitudinal connectivity changes after 6 months of fingolimod treatment were found in cognitive-related RSNs (posterior default mode, left frontoparietal, and orbitofrontal) and were especially evident in specific regions of the posterior default mode network, namely the precuneus and posterior cingulate cortex. These regions are involved in cognitive function and act as network hubs whose connectivity is modulated in relation to several brain networks (42). The significant changes in these regions are also consistent with findings from our previous work, in which we reported a longitudinal fingolimod-related FC decrease between these posterior cortical areas and the cortical area of the right hand (9).

### Evidence of Favorable Cognitive Function Modulation After Fingolimod

Connectivity changes in the posterior default mode network after fingolimod treatment correlated with a PASAT score increase, reflective of an improvement in information processing speed associated with the effects of the drug. In MS, modulation of the default mode network has previously been found to be related to improvement in cognitive tasks after training (43, 44).

Despite the ongoing debate about the adaptive or maladaptive role of functional connectivity changes in the context of MS (45), our finding of decreased FC after fingolimod can be interpreted as a positive phenomenon, in line with previous studies that reported increased functional connectivity in the default mode network associated with poorer performance in cognitive tests (46–48).

One concern is that the observed improvement in PASAT scores and concomitant FC changes of NEDA patients are, at least in part, due to a practice or learning effect. A recent study suggested that short-term practice effects on PASAT are related to brain volume, disease severity, and age and have clinically meaningful prognostic implications. High learners benefited more from fingolimod treatment (49). Therefore, we suggest that an increase in PASAT score, implying cognitive improvement and/or the retention of practice effects, can be interpreted as a positive phenomenon. It should also be noted that PASAT is particularly sensitive to fingolimod treatment (50) and is a difficult and demanding test that is even sensitive to early cognitive changes in patients where room for improvement is still limited (51, 52).

### Study Limitations

One of the main limitations of the present study is the small sample size, along with the lack of a control group of healthy subjects or a group of untreated patients. Although comparison with healthy subjects would have allowed us to characterize the observed FC changes as patient specific, previous studies have shown that MS patients and healthy subjects have different baseline connectivity in most RSNs (53). The longitudinal study design was also meant to compensate for the lack of an untreated MS patient group, which would be unethical. As reported above, baseline clinical and MRI features did not differ between the 2 subgroups in the present study. However, due to the small study sample, we cannot exclude that mechanisms of compensation could take place mainly in patients with less baseline disease activity and less clinical disease burden, as previous studies seem to suggest (32, 34). For the same reason, although we did not find significant subgroup differences in the number of naïve or switching patients (either first- or second-line DMTs), we cannot exclude the relevance of this condition on FC modulation.

Another limitation of the present study is that the anti-inflammatory effects of fingolimod were evaluated only by means of conventional MRI. The use of other techniques, such as positron emission tomography, would have assessed the inflammation of brain tissue outside the lesion areas (54).

A further limitation is the lack of physiological data recordings (e.g., through pulse-oximeter, respiratory bellows, expired gas analyzer) as well as more direct measures of perfusion (e.g., ASL) or vascular reactivity (e.g., breath-holding task). Although the existence of different data-driven methods, physiological noise correction in fMRI data has not emerged as a standard pre-processing step (55), and in the present clinical study, we opted for a more standard pipeline.

The longer-term effects of fingolimod have not been evaluated, and a re-baselining was not performed. Additionally, NEDA outcome is usually evaluated after 1–2 years, and this timeframe would also have allowed us to measure the annualized percentage of brain volume loss and progression to NEDA-4 status. Nevertheless, our percentage of patients with NEDA-3 status at 6 months is consistent with literature. Furthermore, a 6-month observation time was also selected to avoid as much as possible the confounding effects of neurodegeneration (atrophy modifications can take years to occur and brain volume measures acquired in the first period of treatment can be affected by several factors, i.e., pseudoatrophy [see (4)]. Finally, cognitive status was assessed with only the PASAT score, which cannot be considered a global measure of cognitive function. However, the PASAT score has proven its validity in detecting even early cognitive impairment and sensitivity to treatment-related modifications [see (9)].

## Conclusion

The present study showed that in MS, the achievement of NEDA status after disease-modifying treatment may be predicted by higher FC at baseline and is associated with significant brain functional rearrangement after 6 months of therapy. These functional changes occurred in cognitive-related networks and were paralleled by an improvement in information processing speed. FC changes in this initial treatment period may reflect neuroplastic changes promoted by a more efficient functional reserve through the effects of fingolimod. Although the use of fMRI as a tool in clinical decision-making in individual patients seems a long way off, our study suggests that a similar estimate of functional reserve is promising potential predictor of clinical outcome.

## Data Availability Statement

The datasets presented in this article are not readily available because of patient confidentiality and participant privacy restrictions. Requests to access the datasets should be directed to petsas@gmail.com.

## Ethics Statement

This study, involving human participants, was reviewed and approved by the ethics committee of Azienda Policlinico Umberto I, Sapienza University of Rome. The patients/participants provided their written informed consent to participate in this study.

## Author Contributions

CPi contributed to method definition, data analysis, statistics, and manuscript editing. NP contributed to study design, experimental settings, subject recruitment, MRI acquisition, and manuscript editing. LD, ST, and CG contributed to data interpretation and manuscript editing. MC performed MRI acquisition. CPo contributed to study design and recruitment and, together with PP, supervised the study and manuscript editing. All authors contributed to the article and approved the submitted version.

## Conflict of Interest

The dataset used in the present work comes from a previous study (9) that received financial support from Novartis Farma, Italy. NP received speaker fees from Biogen Idec and mission support from Merck Serono, Genzyme, and Novartis. LD received speaking honoraria from Genzyme and Novartis; travel grants from Biogen Idec, Merck Serono, and Teva; and consulting fees from Genzyme, Merck Serono, and Novartis. CPo received consulting and lecture fees from Sanofi-Aventis, Biogen Idec, Bayer Schering, Merck Serono, and Novartis. He also received research funding from Novartis, Sanofi-Aventis, Merck Serono, and Bayer Schering. PP received travel funding from Novartis, Genzyme, and Bracco and speaker honoraria from Biogen Idec. The remaining authors declare that the research was conducted in the absence of any commercial or financial relationships that could be construed as a potential conflict of interest.
