# Supplementary material for: Increased Within-Network Functional Connectivity May Predict NEDA Status in Fingolimod-Treated MS Patients
Source: Front Neurol. 2021 Mar 5;12:632917. doi: 10.3389/fneur.2021.632917 (PMC7973271; doi:10.3389/fneur.2021.632917)
Supplement: Supplementary file 1 [file Table_1.DOCX]

**Supplementary Materials**

Increased within-network functional connectivity may predict NEDA status in fingolimod-treated MS patients

Claudia Piervincenzi^1^, Nikolaos Petsas^2^, Laura De Giglio^3^, Maurizio Carmellini^1^, Costanza Giannì^1^, Silvia Tommasin^1^, Carlo Pozzilli^1,4^, Patrizia Pantano^1,2^

**Affiliations:**

^1^Department of Human Neurosciences, Sapienza University of Rome, Rome, Italy

^2^Department of Radiology, IRCCS NEUROMED, Pozzilli, Italy

^3^Neurology Unit, San Filippo Neri Hospital, Rome, Italy

^4^Multiple Sclerosis Centre, Sant'Andrea Hospital, Rome, Italy

***Address correspondence to:**

Nikolaos Petsas, MD, PhD

Research Associate

IRCSS Neuromed Institute, Pozzilli (IS), Italy

cell: +393289764730; e-mail: petsas@gmail.com

Supplementary Table 1: A list of the image quality metrics (IQMs) extracted from functional MRI data using MRIQC tool (https://github.com/poldracklab/mriqc). Each mean and standard deviation is calculated across all runs of all participants. For information about each IQM please refer to http://mriqc.org.

| **IQM** | **Mean** | **Std. Dev.** | **IQM** | | **Mean** | **Std. Dev.** |
| --- | --- | --- | --- | --- | --- | --- |
| **aor** | 0.001 | 0.002 | **summary bg k** | | 72.975 | 32.606 |
| **aqi** | 0.009 | 0.002 | **summary bg mad** | | 7.7538 | 1.663 |
| **dvars_nstd** | 31.456 | 3.411 | **summary bg mean** | | 49.079 | 6.960 |
| **dvars_std** | 1.049 | 0.040 | **summary bg median** | | 27.946 | 2.247 |
| **dvars_vstd** | 0.984 | 0.017 | **summary bg n** | | 120,219.933 | 5,304.368 |
| **efc** | 0.525 | 0.022 | **summary bg p05** | | 21.394 | 1.457 |
| **fber** | 924.413 | 152.821 | **summary bg stdv** | | 69.872 | 11.843 |
| **fd_mean** | 0.079 | 0.035 | **summary bg p95** | | 152.806 | 36.151 |
| **fd_num** | 7.250 | 9.129 | **summary fg k** | | 1.612 | 0.362 |
| **fd_perc** | 5.179 | 6.521 | **summary fg mad** | | 178.134 | 17.550 |
| **fwhm_avg** | 2.351 | 0.059 | **summary fg mean** | | 899.219 | 44.884 |
| **fwhm_x** | 2.209 | 0.069 | **summary fg median** | | 885.811 | 42.865 |
| **fwhm_y** | 2.561 | 0.075 | **summary fg n** | | 44,286.583 | 4,386.839 |
| **fwhm_z** | 2.283 | 0.067 | **summary fg p05** | | 578.329 | 38.444 |
| **gcor** | 0.027 | 0.016 | **summary fg p95** | | 1,272.078 | 81.524 |
| **gsr_x** | -0.004 | 0.006 | **summary fg stdv** | | 215.050 | 21.991 |
| **gsr_y** | 0.031 | 0.010 | **tsnr** | | 43.476 | 4.803 |
| **snr** | 4.150 | 0.351 | |  |  |  |

Supplementary Table 2: A list of the image quality metrics (IQMs) extracted from structural T1-weighted MRI data using MRIQC tool (https://github.com/poldracklab/mriqc). Each mean and standard deviation is calculated across all runs of all participants. For information about each IQM please refer to http://mriqc.org.

| **IQM** | **Mean** | **Std. Dev.** | **IQM** | **Mean** | **Std. Dev.** |
| --- | --- | --- | --- | --- | --- |
| **cjv** | 0.364 | 0.046 | **summary_csf_k** | 76.131 | 51.585 |
| **cnr** | 3.692 | 0.428 | **summary_csf_mad** | 88.376 | 19.650 |
| **efc** | 0.497 | 0.032 | **summary_csf_mean** | 397.514 | 30.804 |
| **fber** | 6,977.827 | 2,230.668 | **summary_csf_median** | 383.524 | 33.609 |
| **fwhm_avg** | 8.275 | 0.415 | **summary_csf_n** | 346,512.767 | 91,543.911 |
| **fwhm_x** | 4.722 | 0.255 | **summary_csf_p05** | 274.635 | 9.618 |
| **fwhm_y** | 10.624 | 0.590 | **summary_csf_p95** | 538.620 | 37.559 |
| **fwhm_z** | 9.480 | 0.482 | **summary_csf_stdv** | 119.784 | 45.377 |
| **icvs_csf** | 0.255 | 0.015 | **summary_gm_k** | 0.239 | 0.165 |
| **icvs_gm** | 0.385 | 0.013 | **summary_gm_mad** | 46.987 | 4.945 |
| **icvs_wm** | 0.360 | 0.010 | **summary_gm_mean** | 756.924 | 14.172 |
| **inu_med** | 1.448 | 0.066 | **summary_gm_median** | 756.949 | 13.586 |
| **inu_range** | 0.487 | 0.078 | **summary_gm_n** | 150,358.617 | 29,521.438 |
| **qi_1** | 0.000 | 0.000 | **summary_gm_p05** | 677.362 | 16.301 |
| **qi_2** | 0.016 | 0.005 | **summary_gm_p95** | 837.317 | 20.232 |
| **rpve_csf** | 24.974 | 3.367 | **summary_gm_stdv** | 48.540 | 5.346 |
| **rpve_gm** | 15.806 | 1.614 | **summary_wm_k** | 1.170 | 1.170 |
| **rpve_wm** | 19.909 | 2.089 | **summary_wm_mad** | 41.150 | 5.383 |
| **snr_csf** | 3.562 | 1.059 | **summary_wm_mean** | 997.804 | 1.375 |
| **snr_gm** | 15.770 | 1.657 | **summary_wm_median** | 1,000.004 | 0.002 |
| **snr_wm** | 22.587 | 2.478 | **summary_wm_n** | 923,283.317 | 126,028.405 |
| **snr_total** | 13.973 | 1.246 | **summary_wm_p05** | 919.401 | 9.659 |
| **snrd_csf** | 59.139 | 11.744 | **summary_wm_p95** | 1,066.771 | 7.998 |
| **snrd_gm** | 116.250 | 18.855 | **summary_wm_stdv** | 44.814 | 5.067 |
| **snrd_wm** | 153.529 | 24.672 | **tpm_overlap_csf** | 0.221 | 0.012 |
| **snrd_total** | 109.639 | 18.143 | **tpm_overlap_gm** | 0.475 | 0.025 |
| **summary_bg_k** | 43.076 | 46.007 | **tpm_overlap_wm** | 0.544 | 0.025 |
| **summary_bg_mad** | 4.435 | 1.083 | **wm2max** | 0.539 | 0.028 |
| **summary_bg_mean** | 7.620 | 0.885 |  |  |  |
| **summary_bg_median** | 5.997 | 0.957 |  |  |  |
| **summary_bg_n** | 16,235,074.750 | 1,810,822.260 |  |  |  |
| **summary_bg_p05** | 0.000 | 0.000 |  |  |  |
| **summary_bg_p95** | 19.566 | 2.341 |  |  |  |
| **summary_bg_stdv** | 6.838 | 0.884 |  |  |  |
